# Analysing the clinical outcomes between FIGO 2009 and 2023 staging for endometrial cancer—a single institution retrospective study

**DOI:** 10.3332/ecancer.2025.1836

**Published:** 2025-02-05

**Authors:** Divya P Vuppu, Anupama Rajanbabu, Indu R Nair, Anjaly S Nair, Ajay Sasidharan, Kalavagunta Sruthi, Keechilat Pavithran, Reshma Jeladharan

**Affiliations:** 1 Department of Gynaecological Oncology, Amrita Institute of Medical Sciences, Ponekkara Rd, Edappally, Kochi, Ernakulam 682041, Kerala, India; 2 Department of Gynaecological Oncology, James Cook University Hospital, Marton Rd, Middlesbrough TS4 3BW, UK; 3 Department of Pathology, Amrita Institute of Medical Sciences, Ponekkara Rd, Edappally, Kochi, Ernakulam 682041, Kerala, India; 4 Department of Biostatistics, Amrita Institute of Medical Sciences, Ponekkara Rd, Edappally, Kochi, Ernakulam 682041, Kerala, India; 5 Department of Radiation Oncology, Amrita Institute of Medical Sciences, Ponekkara Rd, Edappally, Kochi, Ernakulam 682041, Kerala, India; 6 Department of Medical Oncology, Amrita Institute of Medical Sciences, Ponekkara Rd, Edappally, Kochi, Ernakulam 682041, Kerala, India; a https://orcid.org/0009-0009-8698-4410; b https://orcid.org/0000-0002-2885-8098; c https://orcid.org/0000-0002-4846-4037; d https://orcid.org/0000-0002-5239-7316; e https://orcid.org/0000-0001-7355-8249; f https://orcid.org/0000-003-4639-8256; g https://orcid.org/000-002-6129-5709

**Keywords:** endometrial cancer, staging, prognosis, FIGO 2023

## Abstract

The latest International Federation of Gynaecology and Obstetrics (FIGO) staging for endometrial cancer incorporated molecular profiles and histopathological factors into the basic staging framework. This study was done to determine its implications when applied in real-world clinical practice. The main objectives were to analyse the stage shifts and determine the stage-wise 3-year overall survival (OS) and disease-free survival (DFS) between the two staging systems. It was a single-institution retrospective study that included all Endometrial cancer patients who underwent surgery between January 2015 and June 2021. Cases that underwent primary or incomplete surgery elsewhere and cases with histological types of cancer not included in FIGO 2023 staging were excluded from the study. All patients were staged according to FIGO 2009 and 2023 staging for endometrial cancer. This study found most stage shifts among stage I cases; based on aggressive histology, 33.8% (*n* = 65/192) of cases in stage IA and IB upstaged to stage IIC (2023). Meanwhile, among the advanced stages, most were downstaged, i.e., from stage IIIA to stage IA3 0.6% (*n* = 1/15), from stage IVB to stage IIIB2 (2023), i.e., 32% (*n* = 8/25). This study failed to show any significant differences in the stage-wise 3-year OS rate and DFS rate between the two staging systems. The present study provided preliminary data on the latest FIGO endometrial staging system. Due to the addition of subgroups based on prognostic factors, most upstages occurred in the early stages, while downshifts occurred in the advanced stages. There was no clinical difference in the 3-year OS and DFS rates.

## Introduction

Over the past decade, the availability of data from the Cancer Genome Atlas has made it possible to understand the biological behaviour and characteristics of Endometrial cancer [1]. This data has prompted the much-needed and long-awaited changes in Endometrial cancer staging. After nearly 14 years, the International Federation of Gynaecology and Obstetrics (FIGO) updated the Endometrial carcinoma staging system in 2023. The new staging system incorporates all significant developments in molecular and histological factors that impact the prognosis [2].

The previous staging system was based mainly on anatomic boundaries. Hence, the adjuvant treatment was based on the risk stratification by the European Society of Pathology (ESP), the European Society of Gynaecological Oncology (ESGO) and the European Society for Radiotherapy and Oncology (ESTRO) [3]. However, the new FIGO 2023 staging system has integrated all these molecular and histological parameters, which impact the prognosis and could result in more individualised treatment.

The latest staging system has introduced molecular profiles and histopathological variables, like lymphovascular space invasion (LVSI), to create clinically relevant substages for better prognostication and treatment decisions [2]. However, incorporating the molecular profiles and other changes in the new FIGO 2023 staging for endometrial cancer into real-world clinical practice might be challenging, especially in resource-limited settings. This study was undertaken to understand better the stage shifts with the new staging system and its impact on prognosis compared to FIGO 2009 staging.

## Methods

The study is a single-institute retrospective study conducted in the Department of Gynaecological Oncology and Pathology at the Amrita Institute of Medical Sciences, Kochi, India, between January 2015 and June 2021.

All patients who underwent surgery for endometrial cancer at our institute during this period were enrolled in the study. ‘Patients who underwent surgeries at other hospitals or had histological types not included in FIGO 2023 staging, or only had a biopsy without surgery at our hospital were excluded from the study’. All cases before 2019 with positive LVSI and available slides were reanalysed by a gynaecological oncology pathologist and reclassified into focal or substantial according to WHO classification [4]. When available, molecular data were collected from previous studies on microsatellite instability and polymerase epsilon (POLE) mutation in our institute but were not included in the study, as uniform data were not available.

The electronic medical records were accessed to collect relevant patient data. All patient data were anonymised before analysis. The institute's ethical committee approved the study (IEC-AIMS-2023-GYNEC ONCO-376).

‘Overall survival’ (OS) was defined as the duration between the date of surgery and the last day of follow-up. ‘Disease-free survival’ (DFS) was defined as the duration between the date of surgery and the date of recurrence. All patients were staged according to FIGO 2009 and 2023 staging for endometrial cancer. ‘Upshift’ was defined as any case reclassified to a higher stage or substage, while ‘downshift’ was defined as any case reclassified to a lower stage or substage.

Early stage disease was considered up to stage II, and advanced-stage disease included stages III and IV.

### Patient management

All patients had a preoperative diagnosis based on endometrial biopsy, and imaging, i.e., magnetic resonance imaging or contrast-enhanced computed tomography, was used to assess the extent of the disease. The preferred treatment was minimally invasive staging surgery (Laparoscopy or Robotic), with sentinel lymph node biopsy done for apparently early stage cancers. The sentinel node mapping algorithm of the National Comprehensive Cancer Network was followed for all patients [5]. Infra-colic omentectomy was done in cases of serous, carcinosarcoma and undifferentiated histology on endometrial biopsy reports. Laparotomy was the preferred approach if imaging showed disseminated disease. A multidisciplinary tumour board discussed all cases. The data regarding the adjuvant treatment received by each patient were collected [2].

The adjuvant therapy offered was either observation or vaginal brachytherapy (VBT) +/- external beam radiotherapy (EBRT) in the early stages. Patients in advanced stages received adjuvant chemotherapy and radiotherapy. Concurrent plus adjuvant chemotherapy has been the standard for treating patients in the advanced stage since 2016. From 2020, early stage p53-positive cases received concurrent plus adjuvant chemotherapy.

The surveillance protocol was one visit every 3 months for the first 2 years and one visit every 6 months for the next 3 years. Each visit included a detailed history examination. Imaging annually or when the signs or symptoms were detected during an examination [5, 6].

### Statistical analysis

Statistical analysis was performed using IBM SPSS version 20.0 software (SPSS Inc. Chicago, IL, USA). Categorical variables were expressed using frequencies and percentages. Numerical variables were presented using means and standard deviations. The stage shifts were described using cross tables and visualised using Sankey diagrams. Kaplan Meier survival analysis was used to study the overall and DFS, and the log-rank test was used to compare the survival estimates between stages. A *p*-value of <0.05 was considered statistically significant.

## Results

A total of 381 patients met the inclusion criteria and were enrolled in this study. The mean age of the study population was 59.8 years (±10.7), and the mean body mass index was 28.5 kg/m^2^ (±5.6). Most patients underwent minimally invasive surgery, which included robotic and laparoscopy (79.5%; *n* = 303), out of which most (78%; *n* = 297) underwent robotic surgery. Laparotomy was done in 20.5% (*n* = 78) of cases.

When the histology of the cases was analysed according to FIGO 2009, 74% (*n* = 281) of cases were endometrioid; among them, 58% (*n* = 164), 32% (*n* = 90) and 10% (*n* = 27) were grade 1, 2 and 3, respectively, and 26% (*n* = 100) were non-endometrioid. When analysed according to FIGO 2023, 66.6% (*n* = 254) of cases had non-aggressive histology and 33.4% (*n* = 127) had aggressive histology.

In this study, 77.6% of the 296/381 cases underwent immunohistochemistry (IHC) testing for P53, with 30% identified as having the mutant type. Additionally, doublet IHC testing for mismatch repair deficiency was tested in 30% of the cases. Among these, a loss of PMS2 was observed in 20% of the cases, while a loss of MSH6 was noted in 6%. POLE was tested in 8.6% (33/381) cases as a part of the study, and two were positive.

In the study cohort, 64% (*n* = 243) of patients received adjuvant treatment, with VBT being the most common (Table 1).

Of the overall study cohort of 381 patients, 24.6% (*n* = 94) had stage shifts when reclassified using FIGO 2023. There were 19.4% (*n* = 74) upshifts and 9% (*n* = 17) downshifts (Table 2).

**Figure 1.**
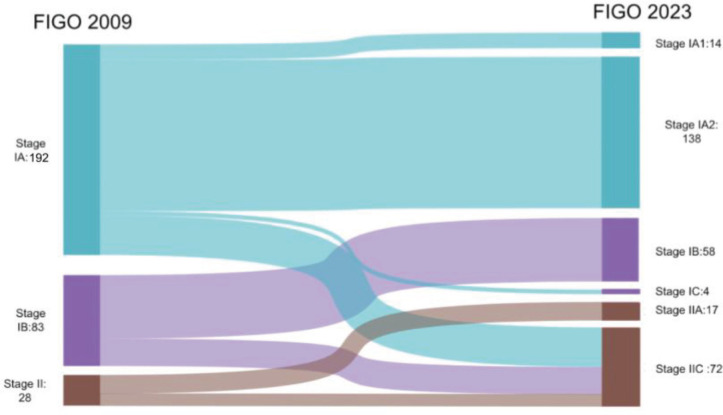
Stage shifts in early stages between FIGO 2009 and 2023.

**Figure 2.**
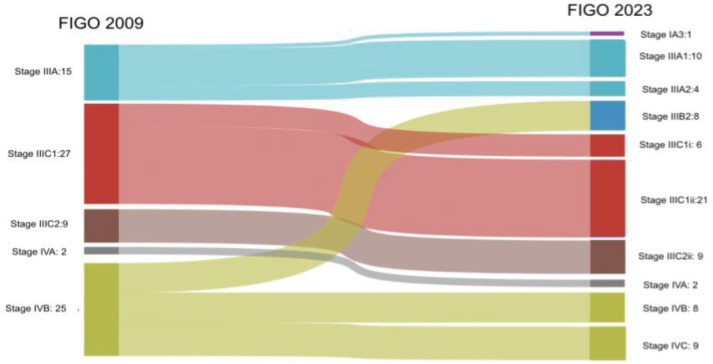
Stage shifts in advanced stages between FIGO 2009 and 2023.

Within stage 1A, 18% (*n* = 36/192) and 30% (*n* = 25/83) of patients from stage 1B were upstaged to stage IIC (2023). Meanwhile, 0.2% (*n* = 4/192) of patients were upstaged to stage IC from stage IA, and 39.3% (*n* = 11/28) of patients from stage II were upstaged to stage IIC. When the advanced stages were analysed, 6.7% (*n* = 1/15) were downstaged to stage IA3 (2023). Among the stage IVB cases, 32% (*n* = 8/25) were downstaged to stage IIIB2 (2023), 32% (*n* = 8/25) were restaged to stage IVB (2023) and 36% (*n* = 9/25) were upstaged to stage IVC (2023) (Figures 1 and 2).

The 3-year OS rate of the 381 patients was 92%, with a median follow-up period of 32 months. The 3-year DFS rate was 86.3%, with a median follow-up period of 31 months.

The stage-wise 3-year OS and DFS to compare the 2009 and 2023 FIGO staging systems were analysed with Kaplein-Meir curves. For stage I cases (2009 versus 2023), the 3-year OS rate was 95.4% versus 95.9%, with HR of 1.1 (0.4–2.9) and *p* = 0.8; for stage II cases, the 3-year

**Figure 3.**
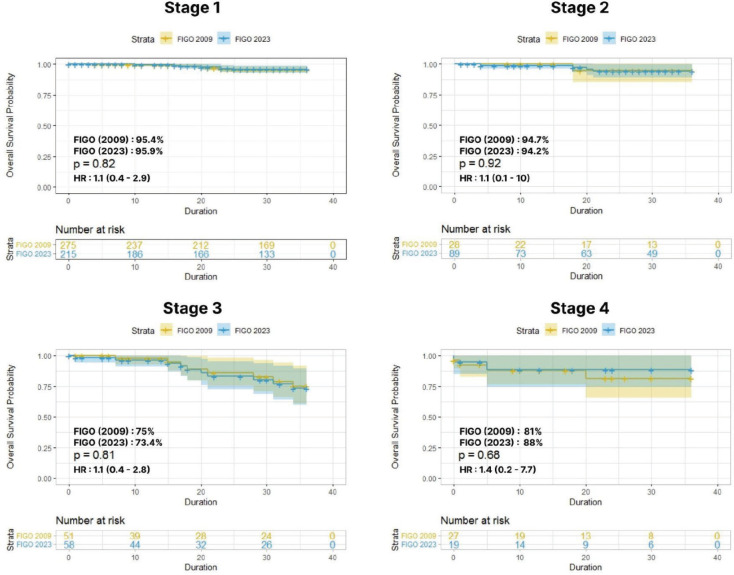
Stage-wise 3-year OS between FIGO 2009 versus 2023.

OS rate was 94.7% versus 94.2%, with HR of 1.1 (0.1–10) and *p* = 0.9. Among the advanced stages, stage III cases had a 3-year OS rate of 75% versus 73.4%, with HR of 1.1 (0.4–2.8) and *p* = 0.8; for stage IV cases, a 3-year OS rate of 81% versus 88% was reported, with HR of 1.4 (0.2–7.7) and *p* = 0.6 (Figure 3).

**Figure 4.**
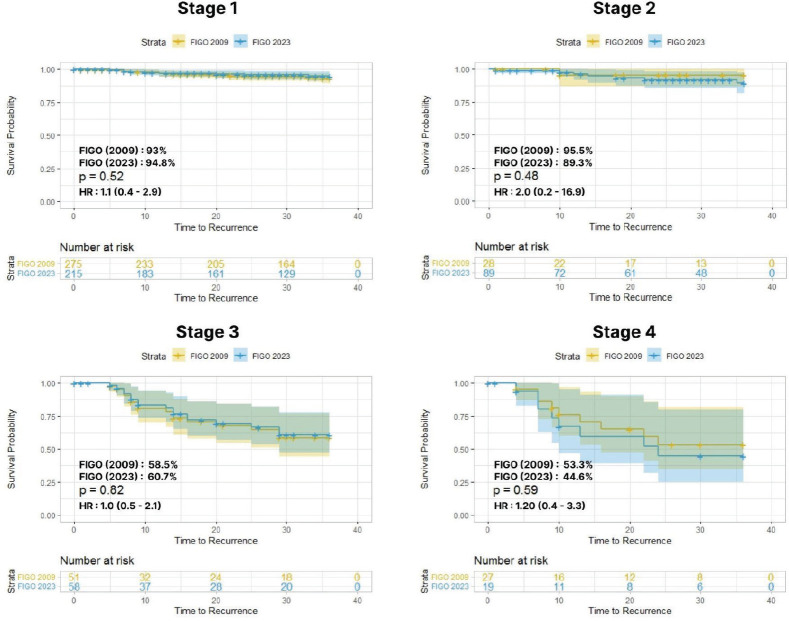
Stage-wise 3-year DFS between FIGO 2009 versus 2023.

The 3-year DFS rate was compared between the two staging systems. For stage I cases, the 3-year DFS rate was 93% versus 94.8%, with HR of 1.1 (0.4–2.9) and *p* = 0.5; for stage II cases, the 3-year DFS rate was 95.5% versus 89.3%, with HR of 2 (0.2–6.9) and *p* = 0.4. Within the advanced stages, stage III cases had a 3-year DFS rate of 58.5% versus 60.7% with HR of 1.0 (0.5–2.1) and *p* = 0.8; among stage IV cases, the 3-year DFS rate was 53.3% versus 44.6%, with HR of 1.2 (0.4–3.3) and *p* = 0.5 (Figure 4).

## Discussion

Most stage shifts happened among stage I cases, as 33.8% (*n* = 65/192) of cases in stage IA and IB were upstaged to stage IC and IIC (2023) based on aggressive histology or p53 mutant status. Whereas, among advanced stages, there was downstaging from stage IIIA to stage IA3 in 0.6% (*n* = 1/15) of cases based on the new criteria for low-grade endometrioid endometrial cancer involving both the endometrium and ovaries. Downstages were also seen from stage IVB to stage IIIB2 (2023), i.e., 32% (*n* = 8/25) and stage IVB (2023), i.e., 32% (*n* = 8/25) for pelvic and abdominal peritoneal metastasis, respectively. This study failed to show any significant difference in the stage-wise 3-year OS rate and DFS rate between the two staging systems.

A handful of studies have analysed the new endometrial staging system (FIGO 2023). In a study by Schwameis *et al* [7], 519 cases were retrospectively analysed among three ESGO-accredited centres for stage shifts between the two staging systems. The researchers found that aggressive histology was one of the main factors for the upstaging, i.e., 23.6% of cases from stage I to stage IIC. These cases also showed a lower 5-year OS (80%) and PFS (87%) than the 2009 staging system. In advanced stages, downshifts were 3.9% in stages IIIB2 and IA3. The researchers found a worse 5-year PFS rate for stage III than the previous staging system (2009) (44.4% versus 54.1%), but statistical analysis on prognosis could not be done because there were fewer cases in some subgroups.

The stage shifts Schwameis *et al* [7] observed were similar to those observed in the current study, in which most upstages occurred in the early stages, while most downshifts occurred in the advanced stages. In another study, Matsuo *et al* [8] analysed 5,473 cases of advanced endometrial cancer stages (stages III-IV) from the National Cancer Institute's Surveillance, Epidemiology and End Results Programme. They found a better 5-year cancer mortality rate in stage 1A3 compared to stage IIIA (11% versus 33%) and a lower 5-year mortality rate in stage IVB compared to stage IIIB2 (42% versus 62%). We could not analyse subgroups, as the numbers of cases and events in some subgroups were insufficient. These two studies validate increased prognostic precision on the new staging system (FIGO 2023).

A retrospective analysis by Senguttan *et al* [9] assessed the prognostic significance of the new staging system (FIGO 2023). The study found a significant difference in the 3-year progression-free interval between cases that remained stage IA and those that were upstaged, with rates of 92.3% and 73%, respectively (*p* = 0.002). However, no significant survival differences were observed in other stages.

Stage I endometrial cancer is a heterogeneous group with varying prognoses. With the introduction of molecular classification, the various distinct subgroups in stage I have been identified and incorporated in the latest FIGO 2023 staging. The new substage of high-risk histology confined to a polyp or endometrium (stage IC) proved to have prognostic value. Stage IC, aggressive histology and P53 mutant expression were associated with lower overall 5-year survival [10].

This study was done in a single institute and with the maintenance of surgeon and pathologist homogeneity. It reveals the potential consequences of using the 2023 staging system without consistent molecular classification, a likely scenario in many developing countries.

Among the study's limitations is a lacuna of LVSI data according to WHO classification in the older data. As this study applied a retrospective analysis, some subgroups had few or no cases, which might have impacted the OS and DFS analysis. Furthermore, the robust use of sentinel lymph node mapping and aggressive treatment with chemotherapy and radiotherapy for all high-grade and non-endometrioid p53-positive tumours might have impacted the DFS and OS data. Even though IHC markers PMS2, MSH6 and P53 were considered in most cases, we could not conduct an analysis based on molecular classifications, as POLE is not routinely done in our institute.

Among the few retrospective studies that have investigated the new FIGO 2023 endometrial cancer staging system, there is no consensus about its impact. Two studies [7, 8] have shown that the new staging system offers greater prognostic accuracy, particularly for cases that were upstaged from IA to IIC and those that were downstaged to IIIB2 or IA3. However, the current study did not find any statistically significant differences in survival outcomes across all stages. This discrepancy suggests that the new system's clinical value may not be fully consistent across all populations and clinical settings. This might be attributed to variations in adjuvant chemotherapy administration or differences in histopathological evaluations, which impact staging. Therefore, it is essential to address these factors, along with ensuring consistency in pathological assessments and equal access to advanced molecular techniques, which are crucial for treatment, prognosis and uniform data collection for research [11]. Without consistent pathological evaluations and access to molecular tools, the benefits of improved staging may not be fully realised.

The global disparity of resources is a significant obstacle to the widespread use of molecular classification [12].

## Conclusion

This study provides initial preliminary data on the latest FIGO endometrial staging system. It showed maximum stage shifts in the early stages due to the introduction of subgroups based on prognostic factors. In the early stages, most cases were upstaged due to aggressive histology (IIC); in the advanced stages, most were downstaged due to pelvic peritoneal disease (IIIB2). The study found no clinical differences in the 3-year OS and DFS rates.

ESGO, ESTRO and ESP are currently revising adjuvant treatment strategies that are aligned with the new prognostic sub-stages. The limited availability of molecular data in resource-poor settings might mask the actual benefits of the new staging system. As with any recent change, time must pass before people fully embrace and adapt to the updated staging system and evaluate its relevance.

## Conflicts of interest

None.

## Funding

No funding was received.

## Author contributions

Study and design: AR, DV Data collection: DV Analysis and interpretation of results: DV, AN Author Draft manuscript preparation: DV, AR, PK, IN All authors reviewed the results and approved the final version of the manuscript.

## Figures and Tables

**Table 1.** Basic demographics.

Mode of surgery	*N*	Per cent
Open	78	20.5
Robotic	297	78
Laparoscopy	6	1.5
Histology	***N***	Per cent
A. Endometrioid	281	74
Grade 1	164	58
Grade 2	90	32
Grade 3	27	10
B. Non-endometrioid	100	26
Histology based on FIGO 2023		
A. Non-aggressive	254	66.6
B. Aggressive	127	33.4
Molecular profile	***N***	Per cent
P53 A. Wild B. Mutant	296 209 87	77.69 70 30
PMS 2 A. Retained B. Loss	113 91 22	29.6 80 20
MSH6 A. Retained B. Loss	121 114 7	31.7 94 6
Adjuvant treatment A. Not received B. Received VBT EBRT+VBT Chemotherapy+VBT Chemoradiotherapy Chemotherapy	138 243 105 39 14 77 8	36 64

**Table 2.** Stage-wise distribution of cases according to FIGO 2009 and 2023.

	FIGO 2009 (*n* = 381)		FIGO 2023 (*n* = 381)
Stage I A	192	Stage IA1	14
		Stage IA2	138
		Stage IA3	1
Stage IB	83	Stage IB	58
		stage IC	4
Stage II	28	Stage IIA	17
		Stage IIB	0
		Stage IIC	72
Stage IIIA	15	Stage IIIA1	10
		Stage IIIA2	4
Stage IIIB	0	Stage IIIB1	0
		Stage IIIB2	8
Stage C1	27	Stage C1 i Stage C1 ii	6 21
Stage C2	9	Stage C2 i Stage C2 ii	0 9
Stage IVA	2	Stage IVA	2
Stage IVB	25	Stage IVB	8
		Stage IVC	9
